# Editorial: Methods and application in fractal analysis of neuroimaging data

**DOI:** 10.3389/fnhum.2024.1453284

**Published:** 2024-07-10

**Authors:** Camillo Porcaro, Stefano Diciotti, Christopher R. Madan, Chiara Marzi

**Affiliations:** ^1^Department of Neuroscience and Padova Neuroscience Center, University of Padua, Padua, Italy; ^2^Institute of Cognitive Sciences and Technologies—National Research Council, Rome, Italy; ^3^Centre for Human Brain Health and School of Psychology, University of Birmingham, Birmingham, United Kingdom; ^4^Department of Electrical, Electronic, and Information Engineering “Guglielmo Marconi” – DEI, University of Bologna, Cesena, Italy; ^5^Alma Mater Research Institute for Human-Centered Artificial Intelligence, University of Bologna, Bologna, Italy; ^6^School of Psychology, University of Nottingham, Nottingham, United Kingdom; ^7^Department of Statistics, Computer Science and Applications “Giuseppe Parenti,” University of Florence, Florence, Italy

**Keywords:** BCI, EEG, fractal analysis, fractal dimension, MRI

Over the past decades, fractal analysis has significantly advanced the approach to understanding complex patterns, marking a substantial departure from conventional analysis methods. Its applications span across several disciplines, with notable implications in the realm of neuroscience (Di Ieva, 2016, 2024), where traditional analyses cannot sufficiently describe the morphological and functional complexity of the human brain. Indeed, fractal analysis, and especially its main index, the fractal dimension (FD), has evolved from describing brain architecture focusing on healthy (Madan and Kensinger, 2016, 2017, 2018; Marzi et al., 2020, 2021, 2024; Pani et al., 2022) and pathological (Marzi et al., 2018; Pantoni et al., 2019) conditions, to a comprehensive tool for analyzing brain functions under physiological (Cottone et al., 2017; Marino et al., 2019; Collantoni et al., 2020; Porcaro et al., 2020b, 2024), pathological conditions (Di Ieva et al., 2014; Smits et al., 2016; Porcaro et al., 2019, 2020a, 2021, 2022a,b,c; Varley et al., 2020; Fiorenzato et al., 2024)—and see Meregalli et al. (2022) and Díaz Beltrán et al. (2024) for in-depth reviews—and brain computer interfaces (BCIs) (Moaveninejad et al., 2024).

This Research Topic provides an overview of the current knowledge on fractal analysis in neuroimaging data through four articles by 37 authors (Total views: 8,719; as of 4 July 2024).

One paper focuses on investigating the transition to dementia in patients with mild cognitive impairment (MCI) and leukoaraiosis using MRI data (Marzi et al.). The longitudinal study involved 64 participants who underwent baseline MRI examinations and annual neuropsychological assessments over 2 years. The transition to dementia was assessed using established diagnostic criteria 2 years after the MRI baseline examination. Using an XGBoost-based machine learning model with Shapley Additive exPlanations (SHAP) values, the FD of the cortical gray matter emerged as the top predictor of dementia transition, highlighting the significant roles of cortical gray and white matter changes in the progression to dementia. The findings suggest that FD is a highly sensitive biomarker for early detection of dementia in MCI patients with leukoaraiosis.

The second paper combines detrended cross-correlation analysis (DCCA) with Riemannian geometry-based classification (RGBC) in the BCI (Racz et al.). DCCA can be used to estimate the covariance structure of electroencephalography (EEG) data, yet computationally expensive in its original form. However, a recently proposed online implementation of DCCA allows for its fast computation and thus makes it possible to employ DCCA in real-time applications such as BCI. In this study, the authors propose to replace the sample covariance matrices (SCMs) obtained from EEG data with the DCCA matrix as input to RGBC and assess its effect on offline and online BCI performance. Combining DCCA with RGBC yields a robust and effective decoder that not only improves upon the SCM-based approach but can also provide relevant information on the neurophysiological processes behind motor imagery in BCI.

In the third paper (D'Andrea et al.), the authors investigated the mindfulness meditation practice, classically divided into focused attention meditation (FAM), and open monitoring meditation (OMM) styles, by high-temporal resolution magnetoencephalography (MEG) data at source-level during FAM, OMM, as well as the complexity and criticality of dynamic transitions between microstates. Results showed that the coverage and occurrence of specific microstates are modulated either by being in a meditative state or performing a specific meditation style. Hurst exponent values in both meditation conditions are reduced with respect to the value observed during rest; Lempel-Ziv complexity showed significant differences between OMM, FAM, and rest MEG condition, with a progressive increase from rest to FAM to OMM.

Last but not least, the final paper (Porcaro et al.) investigates a vital challenge in epilepsy, i.e., to define biomarkers of response to antiseizure medications. Many EEG methods and indices have been developed mainly using linear methods, e.g., spectral power and individual alpha frequency peak (IAF). However, brain activity recorded by EEG is complex and non-linear. In this paper, for the first time, the authors assessed whole EEG brain signal complexity estimated by FD as implemented by Higuchi (1988) to measure the response to antiseizure therapy in patients with focal epilepsy (FE) and compare it with linear methods. Bayes factor analysis showed that FD was 195 times more likely to separate drug-responder patients before (t1, named DR-t1) and after (t2, named DR-t2) the introduction of the antiseizure medications (ASMs) than IAF and 231 times than theta band. This work suggests that FD is a promising measure for monitoring the response to ASMs in FE.

Overall, this Research Topic emphasizes that fractal analysis procedures are more suitable for describing the high complexity of the human brain, quantifying the non-linear, non-stationarity, and non-oscillatory nature of neural activity.

## Author contributions

CP: Writing – original draft, Writing – review & editing. SD: Writing – review & editing. CRM: Writing – review & editing. CM: Writing – original draft, Writing – review & editing.
